# Tirzepatide reduces alcohol drinking and relapse-like behaviours in rodents

**DOI:** 10.1016/j.ebiom.2025.106119

**Published:** 2026-01-07

**Authors:** Christian E. Edvardsson, Louise Adermark, Sam Gottlieb, Safana Alfreji, Thaynnam A. Emous, Yomna Gouda, Annika Thorsell, Milica Vujičić, Cajsa Aranäs, Anna Benrick, Ingrid Wernstedt Asterholm, Marcelo F. Lopez, Howard C. Becker, Elisabet Jerlhag

**Affiliations:** a Department of Pharmacology, Institute of Neuroscience and Physiology, The Sahlgrenska Academy, University of Gothenburg, Gothenburg, Sweden; b Charleston Alcohol Research Center, Center for Drug and Alcohol Programs, Department of Psychiatry and Behavioral Sciences, Medical University of South Carolina, Charleston, USA; c Department of Psychobiology, Paulista School of Medicine (EPM), Federal University of São Paulo (UNIFESP), Sao Paulo, Brazil; d Proteomics Core Facility, The Sahlgrenska Academy, University of Gothenburg, Gothenburg, Sweden; e Department of Physiology, Institute of Neuroscience and Physiology, The Sahlgrenska Academy, University of Gothenburg, Gothenburg, Sweden; f School of Health Sciences, University of Skövde, Skövde, Sweden

**Keywords:** GLP-1, GIP, Alcohol, Ethanol, Reward, Dopamine, Addiction

## Abstract

**Background:**

Alcohol use disorder (AUD) remains a major public health problem, with few effective medications currently available. However, peptides of the gut-brain axis appear to offer promising therapeutic targets for AUD as they influence the mesolimbic reward circuitry.

**Methods:**

Here, we examined the effects of tirzepatide, a long-acting dual glucagon-like peptide-1 receptor (GLP-1R) and glucose-dependent insulinotropic polypeptide receptor (GIPR) agonist approved for diabetes and obesity, using behavioural assays (locomotor activity and conditioned place preference), alcohol intake paradigms (intermittent access two-bottle choice, drinking in the dark and the alcohol deprivation effect), and molecular analyses (microdialysis, electrophysiology and proteomics) in rodents.

**Findings:**

First, tirzepatide effectively attenuated the rewarding properties of alcohol, measured through locomotor stimulation, conditioned place preference, and accumbal dopamine release (P < 0.001). Subsequently, this GLP-1R/GIPR agonist dose-dependently reduced voluntary alcohol consumption (P < 0.001), prevented binge (P < 0.01) and relapse-like drinking (P < 0.001), and maintained efficacy during repeated administration (P < 0.001). Finally, tirzepatide induced sustained synaptic depression in the lateral septum (P < 0.05) and further altered histone regulatory proteins in this region (P < 0.05), suggesting a potential neural substrate for its effects. Moreover, the GLP-1R/GIPR agonist affected metabolic parameters including body weight (P < 0.001), adipose tissue mass (P < 0.01), hepatic triglycerides (P < 0.01) and circulating pro-inflammatory cytokines (P < 0.05).

**Interpretation:**

Together, our findings suggest tirzepatide modulates alcohol-related behaviours through reward-related mechanisms while also affecting physiological consequences associated with long-term alcohol use. Given tirzepatide's established clinical use and the consistency of effects observed here, these results support further investigation for treating AUD and associated complications.

**Funding:**

The study is supported by grants from the 10.13039/501100004359Swedish Research Council (2023-2600, 2020-00559, 2020-01463, 2024-03054), LUA/ALF (723941 & 1005347) from the 10.13039/501100005754Sahlgrenska University Hospital, Alcohol Research Council of the Swedish Alcohol Retailing Monopoly (FO2024-0048), 10.13039/100000002National Institutes of Health (NIH) (P50 AA010761 & U01 AA014095), U.S. Department of Veterans Affairs Office of Research and Development (BLR&D I01BX000813 & IK6BX006299), Herbert & Karin Jacobssons Foundation (2024-Forskning-225), 10.13039/501100014552Adlerbertska Research Foundation (2024-791), Wilhelm & Martina Lundgren’s Research Foundation (2024-SA-4698), Åke Wibergs Foundation (M24-0216), 10.13039/501100008546Swedish Diabetes Foundation (DIA 2024-898) and 10.13039/501100014197Mary von Sydow Foundation (2024-36 & 2024-185). Thaynnam A Emous held an international internship scholarship from the São Paulo Research Foundation (FAPESP), Process Number #2023/18470-5, while conducting research at the University of Gothenburg.

**Research in context**

**Evidence before this study**

Alcohol use disorder (AUD) remains a complex neuropsychiatric condition with substantial personal and societal costs. This complexity appears reflected in current therapeutic approaches, where variable treatment effectiveness, combined with limited new therapeutic options, has constrained advances in care over recent decades. Amid this treatment landscape, glucagon-like peptide-1 receptor (GLP-1R) agonists already established as therapies for metabolic disorders have emerged as potential candidates for addiction treatment, with recent research suggesting they may reduce alcohol consumption in preclinical and clinical studies. Given this momentum, tirzepatide presents an intriguing candidate–a dual glucose-dependent insulinotropic polypeptide (GIP) and GLP-1R agonist with higher binding affinity for GIP receptor (GIPR) than GLP-1R yet distinct GLP-1R signalling properties. This profile appears particularly relevant given recent evidence suggesting that GIPR may influence addiction processes independently as well as synergistically with GLP-1R. Despite established clinical approval for diabetes and obesity, and some anecdotal reports on alcohol consumption, tirzepatide's alcohol-related effects remain unstudied. This knowledge gap appears important given current clinical trials investigating incretin therapies for AUD and its related complications including fatty liver disease and inflammation, areas where tirzepatide shows clinical promise.

**Added value of this study**

Here we provide a systematic evaluation examining tirzepatide's effects across voluntary consumption, binge-like drinking, and relapse paradigms in AUD. Our work shows that tirzepatide effectively reduces these behaviours in both sexes. These reductions occur without signs of nausea/malaise, with effects sustained during repeated administration without tolerance development. Beyond behavioural effects, treatment reduces liver triglyceride content and circulating pro-inflammatory cytokines after prolonged alcohol consumption in rats. We identify the lateral septum as a tentative neural substrate, with proteomic analysis suggesting potential changes in histone-regulatory proteins associated with tirzepatide effects.

**Implications of all the available evidence**

Given tirzepatide's established clinical approval and availability, these findings suggest repurposing an already-approved medication could address one of medicine's persistent treatment challenges.

## Introduction

Alcohol use disorder (AUD) remains a major public health challenge, contributing to substantial morbidity and mortality worldwide.^1^^,^^2^ Despite available treatments, current medications show modest efficacy and are under-prescribed,^3^ highlighting the need for additional effective therapeutic approaches with alternative mechanisms of action. The neurobiological mechanisms underlying AUD involve the mesolimbic dopamine system, where alcohol-induced dopamine release in the nucleus accumbens (NAc) reinforces consummatory behaviours and contributes to the risk of developing AUD later in life.^4^, ^5^, ^6^, ^7^, ^8^ Long-term alcohol exposure leads to persistent neuroadaptations that disrupt mesolimbic system function, contributing to craving and relapse vulnerability.^7^, ^8^, ^9^, ^10^, ^11^, ^12^, ^13^ Research indicates that altered neuroplasticity and epigenetic mechanisms, including histone modifications, maintain these neuroadaptations.^9^, ^10^, ^11^, ^12^, ^13^ This complexity, combined with the limited success of existing therapies, suggests that current treatment approaches may be insufficient. Effective interventions might require strategies that address multiple interconnected systems influencing reward processing and addiction vulnerability.

In this context, gut–brain axis peptides have emerged as promising therapeutic candidates for AUD,^14^, ^15^, ^16^ given not only their apparent capacity to reduce alcohol intake^17^ but also their wide-ranging physiological effects.^18^ The incretin hormones glucagon-like peptide-1 (GLP-1) and glucose-dependent insulinotropic polypeptide (GIP), traditionally recognised for their metabolic functions, also appear to influence central reward processing.^14^, ^15^, ^16^^,^^19^^,^^20^ Preclinical studies demonstrate that GLP-1 receptor (GLP-1R) agonists reduce alcohol consumption, likely by attenuating alcohol's rewarding effects.^21^, ^22^, ^23^, ^24^, ^25^, ^26^, ^27^, ^28^ Early clinical data from randomised trials and observational studies further demonstrates that GLP-1R agonists can reduce alcohol intake in humans.^29^, ^30^, ^31^, ^32^ Building on these findings, clinical trials are now investigating these therapeutic applications more systematically, including studies examining incretin agonists for both alcohol consumption and alcohol-related disorders (ClinicalTrials.gov identifiers: NCT06546384, NCT06409130, NCT07046819, NCT05891587, NCT05895643, NCT06015893, NCT05892432, NCT06939088, NCT06727331, and NCT06994338).

Recent advances have further introduced multi-receptor incretin agonists for diabetes and obesity treatment, with GLP-1R agonism serving as a central component.^20^ Given their apparent multiple modes of action, these compounds may address several aspects of AUD's complex pathophysiology. Among these, tirzepatide, a long-acting dual GLP-1R/GIPR agonist approved for diabetes and obesity, shows enhanced therapeutic outcomes on cardiometabolic diseases compared to selective GLP-1R agonists.^20^^,^^33^^,^^34^ Tirzepatide exhibits higher binding affinity for GIPR than GLP-1R while maintaining distinct GLP-1R signalling properties,^35^ which may be particularly relevant given recent evidence suggesting that GIPR influences addiction processes independently and in combination with GLP-1R.^36^ Tirzepatide's clinical availability presents an opportunity to explore whether dual incretin agonists might offer advantages for AUD treatment. However, whether tirzepatide even affects alcohol consumption, and if so through what mechanisms, remains unexplored.

To address this knowledge gap, we conducted a systematic investigation of tirzepatide's effects across multiple aspects of AUD using preclinical models. Our approach examined tirzepatide's impact on alcohol-related reward processing, voluntary consumption, and binge and relapse-like drinking in both sexes. We also assessed tirzepatide's influence on metabolic and inflammatory parameters, which are often dysregulated in AUD.^37^, ^38^, ^39^ To identify potential neural substrates underlying tirzepatide's effects, we employed electrophysiological recordings across several reward-related brain regions,^9^^,^^40^ including the NAc core and shell, medial prefrontal cortex (mPFC), dorsolateral and dorsomedial striatum (DLS/DMS), and lateral septum (LS). As effects were noticeable only in the LS, we additionally conducted a proteomic analysis of the LS region of brain tissue samples from alcohol-consuming male rats that received repeated tirzepatide treatment. Together, these studies allowed us to evaluate tirzepatide's therapeutic potential while beginning to characterise what biological mechanisms might account for any observed effects.

## Methods

### Animals

Adult male NMRI mice (n = 195, 8–10 weeks old, 25–30 g; Charles River, Sulzfeld, Germany) were used for locomotor activity, conditioned place preference (CPP), microdialysis, food intake, and electrophysiological studies. Binge-like drinking paradigms employed adult male and female C57BL/6J mice (n = 20 per sex, 8–10 weeks old, 25–30 g; Jackson Laboratories, Bar Harbor, ME, USA). Intermittent access two-bottle choice alcohol studies utilised adult male and female Rcc/Han Wistar rats (n = 32 per sex, 8–9 weeks old, 180–250 g; Envigo, Horst, Netherlands), with tissue collected post-mortem for molecular analyses. The three rodent strains were selected based on their established responsivity to alcohol and gut-brain peptides.^22^, ^23^, ^24^^,^^41^ Animals were group-housed upon arrival and acclimated for at least one week under standardised conditions (12/12-h light/dark cycle, 20 °C, 50% humidity) with ad libitum access to standard chow (Harland Teklad Rodent Diet #2916 & 2918, Madison, WI, USA) and water. Animals used for microdialysis, and alcohol intake studies were subsequently single-housed after surgery or at the start of the alcohol baseline period to prevent implant damage and allow for individual consumption measurements. Behavioural and microdialysis experiments were conducted during the light phase when stimulation effects are more pronounced, with 60-min habituation to the testing environment. Alcohol intake studies were performed during dark and light phases for rats and exclusively during the dark phase for mice, when drinking behaviour is heightened. Animals were randomised to equal sized balanced treatment groups. Researchers involved in assessing experimental data were blinded to the treatment conditions. A between-subjects design was used in general, except for two of the alcohol drinking experiments (Exp 7 & 8), which utilised a counterbalanced within-subjects design with one week inter-treatment interval and a two week washout between experiments. Although we conducted each experiment once, this involved multiple testing batches per day and across several days, an approach necessitated by our capacity to test only 4–6 animals simultaneously in certain protocols. Results appeared consistent across all batches and days, which may suggest reliable experimental effects. A few animals/samples were excluded based on pre-established criteria: microdialysis probe misplacement (n = 4), technical/methodological issues (proteomics: 2 samples excluded (the lowest baseline alcohol-consuming male rat in each group) as the maximum number of samples for a proteomics plate were limited to 18, or brain slices in electrophysiology experiment due to no/low response (n = 2)), home-cage fighting in mice (n = 4), or >15% weight loss (n = 0).

### Ethics

All experiments received approval from the Ethics Committee for Animal Research in Gothenburg, Sweden (ethical permits: 4685/23, 3348/20, 3276/20) or the Institutional Animal Care and Use Committee at the Medical University of South Carolina, USA. Studies adhered to the NIH Guide for the Care and Use of Laboratory Animals, ARRIVE guidelines, and 3Rs principle.

### Drugs

For behavioural and neurochemical experiments, alcohol (95% Ethanol, Solveco; Stockholm, Sweden or Warner Graham Co., Cockeysville, MD, USA) was diluted with vehicle (0.9% NaCl) to a 15% (w/v) solution and administered intraperitoneally (IP) at 1.75 g/kg, 5 min prior to testing. Microdialysis experiments required local alcohol administration, achieved by diluting alcohol in modified Ringer's solution (140 mM NaCl, 1.2 mM CaCl_2_, 3.0 mM KCl, and 1.0 mM MgCl_2_, Sigma–Aldrich, Darmstadt, Germany) to 300 mM, corresponding to approximately 50–60 mM outside the probe in the NAc.^42^ These alcohol doses and concentrations were selected for their established ability to stimulate the mesolimbic dopamine system with reproducible effects.^22^, ^23^, ^24^ Alcohol drinking studies employed a 20% (v/v) solution prepared with tap water. Tirzepatide (LY3298176 HCl, MedChemExpress, Sollentuna, Sweden) was dissolved in vehicle (40 mM Tris–HCl, pH 8.0) and administered subcutaneously (SC) 30 min before behavioural testing or alcohol exposure. Dose selection was guided by initial dose–response studies (10, 30, 50, and 70 nmol/kg) that evaluated effects on locomotor activity and food/kaolin intake. While no dose altered baseline 2-h locomotor activity or gross behaviour (Supplementary Figure S1A–D), dose-dependent decreases in food intake and body weight appeared at 24 h without affecting kaolin or water intake (Supplementary Figure S2A–D). Based on these findings, 30 nmol/kg was selected for subsequent experiments to achieve consistent effects 30 min post-administration. The lower dose (10 nmol/kg) was additionally tested in acute alcohol drinking studies to evaluate dose-dependent effects. For technical reasons in the binge-like alcohol drinking experiment tirzepatide was administered IP 1 h before dark onset. To validate this methodological variation, we first compared SC versus IP administration of tirzepatide on ingestive behaviours (Supplementary Figure S2A–D). Both administration routes produced comparable effects.

### Locomotor activity in male mice

**Fig. 1:**
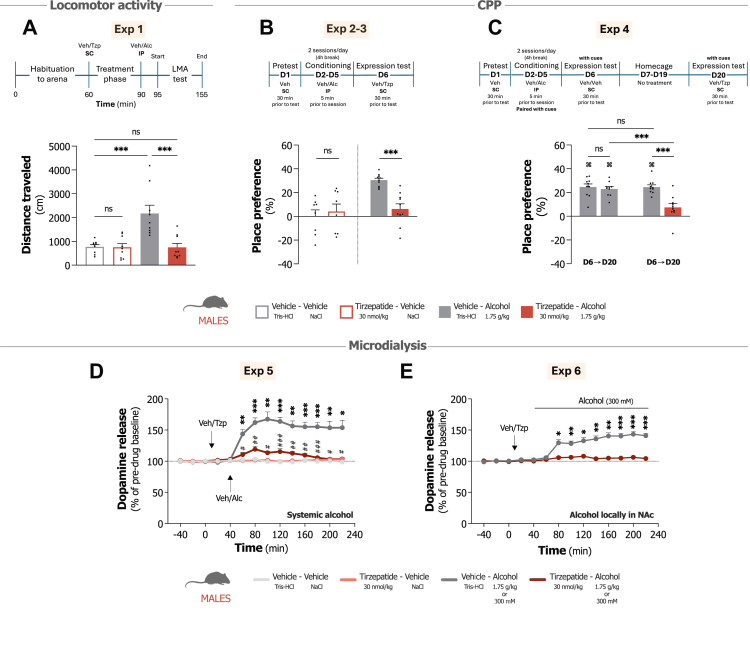
**Impact of tirzepatide on alcohol-related reward behaviours and dopamine release in male mice. A.** Tirzepatide (Tzp; 30 nmol/kg) attenuates alcohol (Alc; 1.75 g/kg, IP)-induced locomotor stimulation (n = 9/group, two-way ANOVA). **B.** Tirzepatide (30 nmol/kg) treatment reduces the expression of alcohol (1.75 g/kg, IP)-induced conditioned place preference (CPP), without affecting CPP itself (n = 8–10/group, unpaired t-test). **C.** Tirzepatide (30 nmol/kg) attenuates alcohol cue-induced CPP following a two-week period in the home cage with no alcohol, cue or context exposure. In contrast, CPP persists in vehicle-treated mice (Veh; n = 10/group, unpaired t-test/independent t-tests). **D.** Tirzepatide (30 nmol/kg) significantly mitigates alcohol (1.75 g/kg, IP)-induced dopamine release in the nucleus accumbens (NAc) shell following systemic alcohol injection (n = 8/group, repeated measures two-way ANOVA). **E.** Tirzepatide similarly blocks dopamine release when we perfused alcohol (300 mM) locally in the NAc (n = 8/group, repeated measures two-way ANOVA). Data show mean ± SEM. ∗P < 0.05, ∗∗P < 0.01, ∗∗∗P < 0.001, ^#^P < 0.05, ^##^P < 0.01, ^###^P < 0.001, ^⌘^P < 0.001 vs pretest.

Horizontal and vertical activity were recorded in six sound-attenuated, ventilated, and dimly lit (3 lx) locomotor boxes (42 × 42 × 20 cm; Open Field Activity System, Med Associates Inc., Georgia, VT, USA). Movement was detected via a two-layered infrared photobeam grid and recorded using Activity Monitor software (Version 7, Med Associates Inc.).^22^, ^23^, ^24^ Male mice (n = 36, 9 per group) underwent 60-min habituation in the test arena before first receiving tirzepatide (30 nmol/kg, SC) treatment and then 30 min later an alcohol (1.75 g/kg, IP) injection (Exp 1). Activity recording began 5 min after the final injection and continued for 60 min. Experimental outline is presented in Fig. 1A.

### Conditioned place preference in male mice

CPP experiments employed four two-chambered arenas (50 × 24 × 24 cm, custom-made, University of Gothenburg, Gothenburg, Sweden) under dim lighting (3 lx), with chambers distinguished by distinct tactile and visual cues.^22^, ^23^, ^24^ The CPP expression protocol (experimental outline is shown in Fig. 1B) was conducted in male mice (n = 20 per experiment, 10 per group), beginning with a 20-min pre-test (day 1) to assess initial place preference following vehicle injection. Conditioning sessions (days 2–5, 20 min each) followed a biased design, pairing alcohol (1.75 g/kg, IP) with the least preferred chamber and vehicle with the preferred chamber. Daily sessions included one alcohol injection and one vehicle injection in a balanced design, alternating between morning and afternoon, with sessions 4 h apart. On expression test day (day 6), mice received tirzepatide (30 nmol/kg, SC) or vehicle 30 min prior to being monitored for place preference for 20 min (Exp 3). A control experiment was conducted to assess tirzepatide's effect on CPP independently of alcohol, following identical procedures but employing vehicle injections in both chambers during conditioning (Exp 2). Moreover, recent evidence suggests that the GLP-1R agonist exenatide reduces alcohol cue reactivity in the NAc and septal regions of individuals with AUD,^30^ prompting us to examine whether tirzepatide exerts comparable effects on cue-induced responses following a period without any exposure to environmental factors that were paired with alcohol. Thus, a third CPP experiment (outlined in Fig. 1C; Exp 4) therefore investigated tirzepatide's influence on cue-induced place preference following a period of no alcohol treatment, nor any cue or context exposure, using the same biased alcohol paradigm with added neutral-valence olfactory cues.^43^^,^^44^ Caraway odour (S-carvone, Sigma–Aldrich) was paired with the alcohol (1.75 g/kg, IP) chamber and mineral oil (Sigma–Aldrich) with the vehicle chamber. Odorants (one drop) were applied to filter paper in perforated plastic tubes positioned at chamber tops. This protocol included a first test day (day 6) where mice received vehicle 30 min before a 20-min test with olfactory cues present. Following two weeks of no treatment in the home cage, mice were divided into equal groups based on first test day results and received tirzepatide (30 nmol/kg, SC) or vehicle 30 min before a second 20-min test (day 20) with olfactory cues present. All experiments were analysed using Observer XT software (Version 15, Noldus, Wagenegen, Netherlands). CPP expression was calculated as the difference in percentage of total time spent in the drug-paired compartment between pre-test and test sessions.

### Microdialysis in male mice

An I-shaped microdialysis probe (20 kDa cut-off membrane with 1 mm exposed length, HOSPAL, Gambro, Sweden) was surgically implanted in the NAc shell four days before experiments as previously described.^22^, ^23^, ^24^ Male mice (n = 52 in total for both experiment 5 and 6, 8–9 per group) were anesthetised with isoflurane (Baxter, Apoteket AB, Gothenburg, Sweden), placed in a stereotaxic frame, and maintained on a heating pad. Local anaesthesia (Xylocaine with adrenaline, 10 mg/ml, 5 μg/ml; Pfizer Inc, Apoteket AB, Gothenburg, Sweden) was applied at the incision site. Carprofen (Rimadyl®, 5 mg/kg, AstraZeneca, Apoteket AB, Gothenburg, Sweden), 0.9% NaCl, and Viscotears were administered for pain management, rehydration, and eye protection. After exposing the skull, holes were drilled for the probe and anchoring screws. The probe was secured with dental cement (DENTALON® Plus, Agntho's AB, Lidingö, Sweden). On experiment days, the probe was connected to a pump and perfused with Ringer's solution at 1.6 μl/min. After a 2-h equilibration period, samples were collected at 20-min intervals throughout the experiment. Following baseline measurements (minutes −40 to 0), tirzepatide (30 nmol/kg, SC) or vehicle was administered at minute 10. Thirty minutes later (minute 40), alcohol was either injected systemically (1.75 g/kg; Exp 5) or perfused through the probe for the remainder of the experiment (300 mM, 40–220 min, Exp 6). Nine additional samples were collected following alcohol exposure. Probe placement was verified histologically using a brain atlas,^45^ and only data from correctly placed probes without haemorrhage were included in analyses (Supplementary Figure S3A and B). Microdialysate samples were analysed using HPLC with electrochemical detection, as described before.^24^ Changes in dopamine and other monoamines and their metabolites were calculated as percentages of the mean of three baseline values before tirzepatide/vehicle treatment. The area under the curve following alcohol exposure (40–220 min) was additionally calculated for further analysis.

### Intermittent access two-bottle choice and the alcohol deprivation effect in male and female rats

**Fig. 2:**
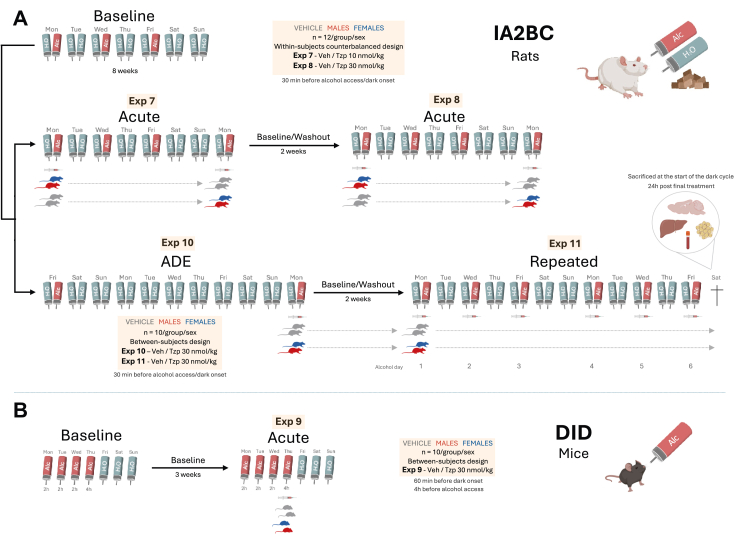
**Experimental outline for the alcohol drinking experiments. A.** Shows the four different alcohol drinking experiments (Exp 7–11) using the intermittent access two-bottle choice (IA2BC) model in male and female rats. **B.** Demonstrates the design for the drinking in the dark (DID) alcohol drinking model, which was employed in male and female mice.

The intermittent access two-bottle choice (IA2BC) paradigm provided male and female rats with alcohol and water access during three 24-h sessions weekly (Monday, Wednesday, Friday), with water-only access on intervening days.^24^^,^^46^ Bottles were switched at dark-phase onset, while food and water remained continuously available. Rats underwent an 8-week baseline period before experimental interventions, during which alcohol, water, and food intake were measured daily and body weight recorded weekly. Following the baseline period, rats were allocated to treatment groups with matched baseline alcohol intake levels. Experimental outline is shown Fig. 2A. During experiments, consumption measurements occurred at 4 and 24 h post-treatment, with corresponding 24-h body weight changes documented. The initial experiments evaluated the acute effect of different doses of tirzepatide (10 nmol/kg, SC; Exp 7 or 30 nmol/kg, SC; Exp 8) on alcohol intake in male and female rats (n = 24, 12 per group), using counterbalanced within-subjects design, with tirzepatide or vehicle administered 30 min prior to alcohol access/dark onset. To assess tirzepatide's impact on relapse-like behaviour, we utilised the alcohol deprivation effect (ADE) model^25^^,^^47^ in a separate batch of male and female rats (n = 40, 10 per group) This protocol involved an 8-week baseline alcohol consumption period followed by 10-day alcohol deprivation within the intermittent access paradigm. Rats then received single tirzepatide (30 nmol/kg, SC) or vehicle administration 30 min before alcohol reintroduction/dark onset, with relapse-like drinking quantified as percentage change from baseline intake (Exp 10). Another experiment in this batch of rats examined repeated tirzepatide (30 nmol/kg, SC) or vehicle administration effects spanning six alcohol drinking days across two weeks following the baseline period (Exp 11). Tirzepatide or vehicle were always administered 30 min prior to alcohol access/dark onset. This design allowed assessment of treatment efficacy over an extended timeframe. Upon completing the repeated administration study, rats were euthanised, 24 h after the final treatment, following a full day of alcohol access. Brains were rapidly removed, flash-frozen, and stored at −80 °C for subsequent analysis. We additionally dissected and weighed several metabolically relevant tissues: gastrocnemius muscle, intrascapular brown adipose tissue (iBAT), subcutaneous inguinal white adipose tissue (sWAT), gonadal white adipose tissue (gWAT), retroperitoneal and perirenal white adipose tissue (rpWAT), and liver. The liver's middle lobe was flash-frozen and stored at −80 °C. Trunk blood was also collected using serum tubes (Z-gel tubes with clotting activator, Sarstedt, Germany) and stored at −80 °C for later analysis.

### Drinking in the dark in male and female mice

A standardised drinking in the dark (DID) protocol in adult male and female mice (n = 40, 10 per group) was used to examine binge-like alcohol drinking.^48^ The experimental design (outlined in Fig. 2B) utilised repeated four-day alcohol access cycles, where days 1–3 involved 2-h sessions and day 4 extended to 4 h as the primary test day. Three days without alcohol separated each cycle. Sessions started 3 h after dark-phase onset, with food remaining available throughout while water was temporarily removed during alcohol access periods. For the test, mice received either vehicle or tirzepatide (30 nmol/kg, IP) 1 h before dark onset, followed by 4-h alcohol access during the dark phase (Exp 9).

### Post-mortem measurements of liver triglycerides and serum cytokines in alcohol-consuming male and female rats

Liver tissue samples from the repeated alcohol drinking experiment (Exp 11) were processed for triglyceride analysis using standard lipid extraction techniques. Tissue was lysed in 2:1 chloroform:methanol and washed with 0.9 M NaCl to achieve phase separation, as previously described.^49^ The triglyceride-containing lower phase was collected and evaporated overnight. Dried triglyceride pellets were resuspended in isopropanol (Sigma Aldrich) and quantified using a commercial triglyceride kit (Randox Laboratories Ltd, Crumlin, UK) according to manufacturer's instructions. Absorbance was measured at 500 nm with 546 nm correction using a Spectramax i3x multiplate reader (Molecular Devices, San Jose, CA, USA). Serum cytokine levels were also measured from blood samples from the repeated alcohol drinking experiment (Exp 11) using a custom-ordered Bio-Plex Pro™ rat Cytokine Assay kit (10014905, Bio-Rad, Hercules, CA, USA) to quantify five cytokines, based on the literature^50^^,^^51^: interleukin (IL)-1β, IL-6, IL-10, tumour necrosis factor alpha (TNFα), and monocyte chemoattractant protein-1 (MCP-1) using the Bio-Plex 200 system (Bio-Rad) according to manufacturer's instructions.

### Electrophysiological recordings in alcohol-naïve male mice

To identify target regions for subsequent brain tissue analyses, we first used electrophysiological screening to determine if any reward-related circuits^9^^,^^40^ would demonstrate sustained neuroplasticity following acute tirzepatide treatment (Exp 12). In alcohol-naïve male mice (n = 10), ex vivo neural activity was evaluated 24 h after a single administration of tirzepatide (30 nmol/kg, SC) across several brain regions, including the NAc core and shell, mPFC, DLS, DMS, and LS (as shown in Fig. 5). This experiment guided the selection of brain region for the proteomic analysis in samples from the long-term alcohol-drinking rats that received repeated tirzepatide treatment (Exp 11). Brain slices were prepared 24 h after tirzepatide or vehicle administration, as previously described.^24^^,^^52^ In brief, brains were removed and submerged in ice-cold modified artificial cerebrospinal fluid (aCSF) cutting solution (220 mM sucrose, 2 mM KCl, 6 mM MgCl_2_, 26 mM NaHCO_3_, 1.3 mM NaH_2_PO_4_, 0.2 mM CaCl_2_, and 10 mM d-glucose; Sigma–Aldrich, Darmstadt, Germany), continuously bubbled with a gas mixture of 95% O_2_/5% CO_2_. Brains were sectioned coronally at 300 μm using a vibratome to obtain slices containing regions of interest. The slices were transferred to a custom-made incubation chamber with aCSF (124 mM NaCl, 4.5 mM KCl, 2 mM CaCl_2_, 1 mM MgCl_2_, 26 mM NaHCO_3_, 1.2 mM NaH_2_PO_4_, and 10 mM d-glucose, with a sucrose-adjusted osmolarity of 315–320 mOsm, Sigma–Aldrich) and continuously bubbled with 95% O_2_/5% CO_2_. The slices were incubated in aCSF for 30 min at 30 °C and then at room temperature for the remainder of the day. Slices were then placed in the recording chambers for field-potential recordings. Field potentials were evoked using a stimulation electrode positioned near (0.2–0.3 mm) the recording electrode in each brain region. Population spike (PS) amplitudes were evoked using seven-step increasing stimulation protocols. Paired-pulse stimulation (50 ms interpulse interval, 0.1 Hz) was used to calculate paired-pulse ratio (PPR, PS2/PS1), to estimate changes in the probability of transmitter release. Data were acquired using Clampfit 10.2 software (Molecular Devices, Foster City, CA, USA).

### Proteomics - global relative quantification - analysis of brain samples from alcohol-consuming male rats

Building on electrophysiological findings that identified the LS as a region exhibiting sustained responses to tirzepatide, we performed a global quantitative proteomic analysis of LS tissue from alcohol-consuming male rats subjected to repeated tirzepatide administration (Exp 11). The LS was microdissected using a brain-slicing matrix on dry ice, weighed, and stored at −80 °C until analysis as previously described.^24^ Protein extraction employed lysis buffer (2% sodium dodecyl sulphate, 100 mM triethylammonium bicarbonate) with a Covaris ML230 ultrasonicator. Protein concentrations were determined using the Pierce BCA Protein Assay Kit (Thermo Scientific, Gothenburg, Sweden). Sample processing followed a modified SP3 method. Samples and references (40 μg) underwent reduction (100 mM DTT), alkylation (20 mM iodoacetamide), and precipitation on Sera-Mag™ SpeedBeads (Cytiva, Uppsala, Sweden) using ethanol. After washing and drying, beads were resuspended in 100 mM TEAB for protein digestion with Trypsin/Lys-C mix (1:25) for 2 h, followed by trypsin (1:50) overnight. Following bead removal, peptide concentrations were determined using Pierce™ Quantitative Fluorometric Peptide Assay (Thermo Scientific). Peptide samples (20 μg) were labelled using TMT pro 18-plex isobaric mass tagging reagents (Thermo Fisher Scientific), pooled into one TMT-set, and purified using HiPPR Detergent Removal Resin and Pierce™ Peptide Desalting Spin Columns. The TMT-set underwent basic reversed-phase chromatography (bRP-LC, pH10) fractionation into 36 fractions over 70 min. Mass spectrometry analysis employed an Orbitrap Eclipse Tribrid mass spectrometer with FAIMS Pro ion mobility system interfaced with an nLC 1200 liquid chromatography system. Peptides were separated on a C18 35 cm column over 90 min, with data acquired using SPS MS3 methodology. Raw files were processed using Proteome Discoverer (Ver 3.0, Thermo Scientific) against UniProt Swiss-Prot *Rattus norvegicus* database using Sequest search engine. Only unique peptides were used for relative quantification, with proteins required to pass a 5% false discovery rate threshold. Proteins showing significant expression changes were cross-referenced with existing literature and the UniProtKB database^53^ to identify those linked to histone and chromatin processes (complete reference list in Supplementary Table S1).

### Statistics

Statistical analyses were performed using GraphPad Prism (version 10.4.1, GraphPad Software Inc., Boston, MA, USA). The statistical approach and sample size selection was as described previously.^24^ In brief, group sizes were chosen according to 3Rs principles and informed by prior experience. Sample size calculations assumed a 5% significance level, 80% power, and two-tailed testing. Based on prior experience, we anticipated detecting mean differences of 1.0–1.5 g/kg in 24 h alcohol intake with estimated standard deviations of 0.7–0.9 g/kg (Cohen's d ≥ 0.8), indicating a minimum of seven animals per group would be sufficient, aligning with previous work.^24^^,^^25^ Normal distribution was assessed using the Shapiro–Wilk test. All subsequent tests were two-tailed with significance threshold at p < 0.05. For comparisons between two groups in behavioural, intake, or electrophysiology experiments, paired or unpaired Student's t-tests or independent t-tests were applied as appropriate. Comparisons among three or more groups employed one-way or two-way ANOVA with Bonferroni or Fisher's LSD post-hoc tests respectively. For experiments with repeated measures (microdialysis, repeated alcohol drinking, and electrophysiology), repeated-measures two-way ANOVA with Bonferroni post-hoc tests were utilised. Welch's t-test was used on log2-transformed data to identify DEPs. Proteins with a P-value < 0.05 and fold-change ≥ 10% (log2 fold-change ≤ −0.137 or ≥ 0.137) were considered as differentially expressed. All data are presented as mean ± standard error of the mean (SEM) with individual values shown when appropriate.

### Role of funders

The funding sources did not play a role for the study design, collection, analysis, and interpretation.

## Results

### Tirzepatide disrupts alcohol-induced behaviours and accumbal dopamine release in male mice

Alcohol consumption activates the mesolimbic dopamine system,^6^ producing locomotor stimulation, CPP, and dopamine release in the NAc. These behavioural and neurochemical changes underlie alcohol's rewarding properties and contribute to the risk of developing AUD.^5^, ^6^, ^7^, ^8^ Given tirzepatide's potential to influence reward processing, we first tested whether acute tirzepatide administration (30 nmol/kg, SC) could alter these alcohol-induced (1.75 g/kg, IP) reward responses in male mice.

We first examined alcohol-induced locomotor stimulation (Exp 1). Tirzepatide alone had no effect on baseline locomotion compared to vehicle (P = 0.950), while alcohol produced the expected locomotor activation (P < 0.001) (treatment F_1,32_ = 11.84, P = 0.002, interaction F_1,32_ = 11.23, P = 0.002; Fig. 1A). Tirzepatide pretreatment significantly blunted this alcohol-induced stimulation (P < 0.001). In fact, locomotor activity in mice receiving both tirzepatide and alcohol was virtually indistinguishable from vehicle-only controls (P = 0.991). We next examined tirzepatide's influence on alcohol CPP. A control experiment (Exp 2) verified that tirzepatide itself did not affect place conditioning when vehicle was paired with both compartments (t_14_ = 0.49, P = 0.631; Fig. 1B). When alcohol was paired with one side, vehicle-treated mice developed clear preference (P < 0.001) for the alcohol-associated environment (Exp 3). Tirzepatide treatment markedly reduced this preference (t_18_ = 5.23, P < 0.001; Fig. 1B). This guided us to examine another clinically relevant question. Recent evidence has shown that GLP-1R agonist exenatide reduces alcohol cue reactivity in NAc and septal regions in patients with AUD,^30^ leading us to test whether tirzepatide might affect cue-induced place preference after prolonged abstinence. For this experiment (Exp 4) we incorporated both environmental and olfactory cues in the CPP paradigm along with a 14-day period in the homecage without any exposure to alcohol, experimental context or cues. While vehicle-treated mice retained strong preference (P < 0.001) for alcohol-associated contexts and cues on day 20, tirzepatide treatment substantially reduced this preference on the last test day (t_18_ = 4.13, P < 0.001; Fig. 1C).

These behavioural findings across locomotion, CPP, and cue-induced preference suggested that tirzepatide affects central reward processing mechanisms. We therefore employed microdialysis to investigate the neurochemical basis underlying these effects, examining alcohol-induced dopamine release in the NAc across two experimental paradigms. Systemic alcohol administration (Fig. 1D) produced pronounced elevation in NAc dopamine release compared to vehicle (treatment F_3,28_ = 57.47, P < 0.001, interaction F_39,364_ = 17.42, P < 0.001). Tirzepatide pretreatment substantially reduced this alcohol-induced dopamine response (Exp 5). Area under the curve analysis confirmed that tirzepatide alone did not affect baseline dopamine release (P > 0.999), with no significant differences between vehicle controls and tirzepatide-alcohol treated mice (P = 0.162). This effect moreover extended to alcohol-induced increases in dopamine metabolites 3, 4-dihydroxyphenylacetic acid (DOPAC), 3-methoxytyramine (3-MT), and homovanillic acid (HVA), which tirzepatide similarly reduced (Supplementary Figure S4A–C). We also detected alterations in noradrenergic and serotonergic transmission (Supplementary Figure S4D–G), though these changes appeared less pronounced than the dopaminergic effects (Supplementary Figure S4H). To explore if these findings reflected tirzepatide's influence on NAc dopamine responses specifically, rather than indirect systemic effects, we perfused alcohol locally through the probe (Fig. 1E). Local alcohol application (Exp 6) evoked robust dopamine increases that systemic tirzepatide administration significantly attenuated (treatment F_1,14_ = 61.52, P < 0.001, interaction F_13,182_ = 25.12, P < 0.001), suggesting tirzepatide can modulate alcohol's dopaminergic effects within the reward circuitry itself.

### Acute tirzepatide treatment dose-dependently reduces alcohol consumption in male and female rats

To further assess tirzepatide's effectiveness across different alcohol drinking phenotypes and potential sex-specific effects, we conducted complementary experiments in male and female rodents. These experiments evaluated tirzepatide's effects on voluntary alcohol consumption across multiple established paradigms: the IA2BC,^46^ DID,^48^ and ADE^47^ models.

**Fig. 3:**
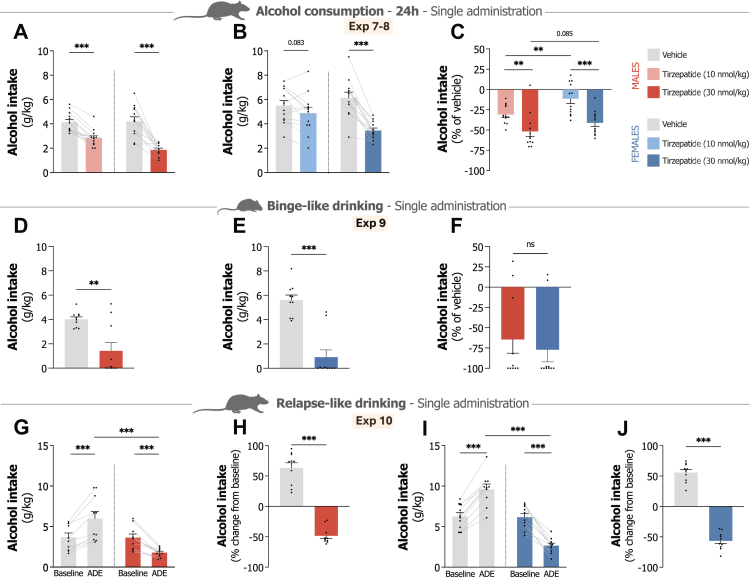
**Effects of single administration of tirzepatide on alcohol intake, binge-like drinking and relapse-like drinking in male and female rodents. A.** Single tirzepatide administration (10 and 30 nmol/kg) significantly reduces 24-h alcohol intake in male rats compared to vehicle (n = 12, paired t-test). **B.** Female rats also significantly reduced 24-h alcohol consumption following single tirzepatide treatment 30 nmol/kg but only showed a trend with the 10 nmol/kg dose (n = 12, paired t-test). **C.** Percent reduction in alcohol intake relative to vehicle demonstrates dose and sex-related effects, with the effect more pronounced in the higher (30 nmol/kg) compared to the lower (10 nmol/kg) dose as well as in males compared to females (n = 12/group, two-way ANOVA). **D.** Single tirzepatide administration (30 nmol/kg) significantly attenuates binge-like drinking in male mice (n = 10/group, unpaired t-test). **E.** Female mice also exhibit significant reduction in binge-like alcohol consumption following single tirzepatide treatment (30 nmol/kg; n = 10/group, unpaired t-test). **F.** Percent comparison against vehicle demonstrates comparable tirzepatide effectiveness (30 nmol/kg) on binge-like drinking between sexes with no statistically significant difference between males and females (n = 10/group, unpaired t-test). **G.** Vehicle-treated male rats exhibit significantly elevated alcohol intake during post-deprivation sessions compared to baseline consumption levels, whereas tirzepatide administration (30 nmol/kg) effectively blocks this relapse-like drinking behaviour (n = 10/group, two-way ANOVA). **H.** Percent change from baseline in males demonstrates significant attenuation of relapse-like drinking behaviour by tirzepatide (30 nmol/kg; n = 10/group, two-way ANOVA). **I.** Vehicle-treated females demonstrate significantly elevated alcohol intake in post-deprivation sessions compared to baseline, whereas single-dose tirzepatide administration (30 nmol/kg) inhibits this relapse-like alcohol drinking effect and further reduces alcohol intake compared to baseline levels (n = 10/group, two-way ANOVA). **J.** Percent change from baseline in females shows tirzepatide efficacy (30 nmol/kg) in preventing relapse-like drinking (n = 10/group, two-way ANOVA). Data show mean ± SEM. ∗P < 0.05, ∗∗P < 0.01, ∗∗∗P < 0.001.

We first assessed acute tirzepatide effects using two doses (10 and 30 nmol/kg, SC) versus vehicle control on alcohol intake in the intermittent access paradigm to examine potential dose-dependent effects in both sexes (Exp 7 and 8). In male rats, the lower dose (10 nmol/kg) showed minimal impact on alcohol, water, or food intake at 4 h (Supplementary Figure S5A–E). By 24 h, this dose significantly reduced alcohol intake (t_11_ = 7.81, P < 0.001; Fig. 3A), alcohol preference (t_11_ = 2.43, P = 0.033), food consumption (t_11_ = 6.07, P < 0.001), and body weight (t_11_ = 9.60, P < 0.001), while water intake remained unchanged (Supplementary Figure S5F–J). The higher dose (30 nmol/kg) produced more pronounced effects, decreasing alcohol intake (t_11_ = 4.47, P = 0.001), alcohol preference (t_11_ = 2.64, P = 0.023), and food consumption (t_11_ = 4.75, P < 0.001) while not significantly alter water intake (t_11_ = 2.04, P = 0.066) at the 4-h timepoint (Supplementary Figure S5K–O). At 24 h, this dose significantly reduced alcohol consumption (t_11_ = 6.14, P < 0.001; Fig. 3A), alcohol preference (t_11_ = 4.25, P = 0.001), food intake (t_11_ = 18.90, P < 0.001) and body weight (t_11_ = 15.30, P < 0.001), while elevating water intake (t_11_ = 3.24, P = 0.008; Supplementary Figure S5P–T). Direct comparison indicated that 30 nmol/kg produced a greater reduction in alcohol consumption (−51.7 ± 6.3%) compared to the 10 nmol/kg dose (−30.9 ± 3.3%; t_44_ = 2.93, P = 0.005), confirming dose-related effects (Fig. 3C).

Parallel studies in female rats revealed similar dose-dependent responses. The lower tirzepatide dose (10 nmol/kg) produced minimal changes in alcohol, water, or food intake at 4 h (Supplementary Figure S6A–E). At 24 h, we observed no significant effect on alcohol intake (t_11_ = 1.91, P = 0.083; Fig. 3B), but noticed significant decreases in food consumption (t_11_ = 3.78, P = 0.003) and body weight (t_11_ = 2.92, P = 0.014), while alcohol preference and water intake remained unaffected (Supplementary Figure S6F–J). The higher dose (30 nmol/kg) in females did not significantly reduce alcohol intake (t_11_ = 1.65, P = 0.128) or alcohol preference (t_11_ = 2.01, P = 0.070) at 4 h, nor change food intake, but increased water intake (t_11_ = 2.71, P = 0.020) (Supplementary Figure S6K–O). At 24 h, this dose significantly reduced alcohol consumption (t_11_ = 6.96, P < 0.001; Fig. 3B) and alcohol preference (t_11_ = 6.41, P < 0.001) diminished food intake (t_11_ = 10.24, P < 0.001) and body weight (t_11_ = 7.12, P < 0.001), while increasing water intake (t_11_ = 4.39, P = 0.001; Supplementary Figure S6P–T). Direct dose comparison in females showed significantly greater alcohol intake suppression with the 30 nmol/kg dose (−41.3 ± 4.3%) compared to the 10 nmol/kg dose (−11.7 ± 5.7%; t_44_ = 4.15, P < 0.001), suggesting dose-related effects (Fig. 3C). While the effect on alcohol intake differed between the sexes with the 10 nmol/kg dose, males (−30.9 ± 3.3%) and females (−11.7 ± 5.7%; t_22_ = 3.30, P = 0.003; Fig. 3C), we noticed no significant sex differences with the 30 nmol/kg dose on alcohol intake between males (−51.7 ± 6.3%) and females (−41.3 ± 4.3%; t_22_ = 1.80, P = 0.085; Fig. 3C). Alcohol consumption also returned to baseline levels 48 h post-treatment in both sexes (Supplementary Figure S7A–B), indicating no apparent signs of alcohol taste aversion and that the suppressive effect of an acute tirzepatide injection on alcohol intake did not extend beyond this timeframe.

### Tirzepatide attenuates binge-like drinking in male and female mice

Given that binge-like alcohol consumption is a clinically relevant concern,^54^ we tested whether tirzepatide affects this drinking phenotype in male and female mice using the DID paradigm (Exp 9). Mice received either vehicle or tirzepatide (30 nmol/kg, IP) 1 h before dark onset, followed by 4-h alcohol access during the dark phase. Both male (t_18_ = 3.66, P = 0.002; Fig. 3D) and female mice (t_18_ = 6.41, P < 0.001; Fig. 3E) showed significant reductions in binge-like drinking, with no significant sex differences in efficacy: males (64.5 ± 16.9%) versus females (77.2 ± 14.9%) relative to vehicle (t_18_ = 0.56, P = 0.581; Fig. 3F).

### Tirzepatide prevents relapse-like drinking in male and female rats

As preventing relapse remains one of the most persistent challenges in AUD treatment,^9^^,^^55^^,^^56^ we next tested whether tirzepatide could also affect relapse-like drinking behaviour. Using the ADE model, which captures the temporary increase in alcohol consumption following forced abstinence,^47^ we assessed tirzepatide's (30 nmol/kg, SC) acute impact on this relapse-like response (Exp 10).

Following 10 days of alcohol deprivation, vehicle-treated male rats developed the expected ADE, with consumption rising significantly above baseline levels (P < 0.001) (treatment F_1,18_ = 8.29, P = 0.010, interaction F_1,18_ = 58.40, P < 0.001; Fig. 3G). Tirzepatide treatment blocked this response entirely. Rather than showing increased drinking, treated rats reduced alcohol intake compared to baseline (P < 0.001). Direct comparison revealed substantial between-group differences: tirzepatide-treated males showed 48.3 ± 4.4% reduction from baseline while vehicle-treated counterparts increased consumption by 63.5 ± 8.3% (t_18_ = 11.90, P < 0.001; Fig. 3H). Additionally, tirzepatide increased water intake while reducing alcohol preference, food consumption and body weight (Supplementary Figure S8A–E).

Female rats showed comparable responses. Vehicle-treated females developed robust ADE (P < 0.001) (treatment F_1,18_ = 31.56, P < 0.001, interaction F_1,18_ = 177.40, P < 0.001; Fig. 3I), whereas tirzepatide treatment again prevented the rebound response and actually decreased alcohol consumption below baseline (P < 0.001). Tirzepatide produced substantial prevention in females as well, with 56.5 ± 4.6% reductions compared to 55.7 ± 4.9% increases in vehicle controls (t_18_ = 16.77, P < 0.001; Fig. 3J). As in males, tirzepatide also increased water intake while decreasing alcohol preference, food consumption and body weight (Supplementary Figure S8F–J).

### Sustained effect of tirzepatide in reducing alcohol consumption with repeated administration in male and female rats

To address whether tirzepatide maintains effectiveness during repeated administration, an important consideration for any potential addiction therapy,^57^ we examined the effects of repeated tirzepatide (30 nmol/kg, SC) or vehicle administration across six alcohol drinking days spanning two weeks following the baseline period (Exp 11).

**Fig. 4:**
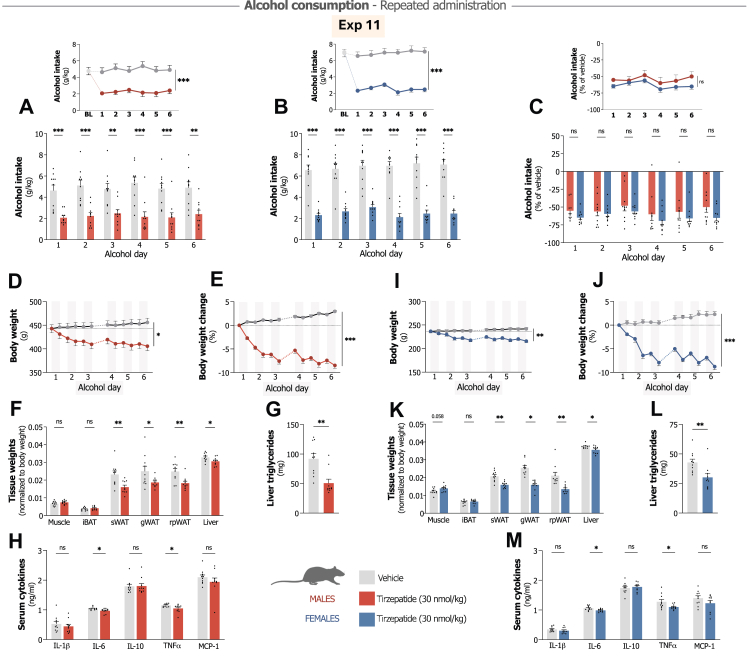
**Effects of repeated tirzepatide administration on alcohol consumption, body composition and inflammation parameters in male and female rats.** All experiments used n = 10/group with repeated tirzepatide (30 nmol/kg) or vehicle treatment. **A.** Tirzepatide significantly attenuates alcohol intake in male rats on all alcohol days compared to vehicle group, which shows similar baseline (BL) alcohol intake levels (repeated measures two-way ANOVA). **B.** Female rats show comparable reduced alcohol intake following tirzepatide administration, with consistent efficacy throughout all alcohol days (repeated measures two-way ANOVA). **C.** Alcohol intake relative to vehicle demonstrates comparable reducing effects across sexes with no statistically significant sex differences (repeated measures two-way ANOVA). **D-E.** Tirzepatide reduces body weight and percent body weight change in male rats compared to vehicle (repeated measures two-way ANOVA). **F.** Post-mortem tissue analysis in males shows significant reductions in subcutaneous inguinal (sWAT), gonadal (gWAT), and retroperitoneal (rpWAT) white adipose tissues and liver weight, while muscle (gastrocnemius) and intrascapular brown adipose tissue (iBAT) remain unaffected (unpaired t-tests). **G.** Tirzepatide significantly reduces hepatic triglyceride content in alcohol-drinking males (unpaired t-test). **H.** Treatment decreases interleukin (IL)-6 and tumour necrosis factor alpha (TNFα) serum levels, while IL-1 beta (β), IL-10 and monocyte chemoattractant protein-1 (MCP-1) remain unaffected (unpaired t-test). **I-J.** Female rats exhibit decreased body weight and percent body weight change following tirzepatide treatment (repeated measures two-way ANOVA). **K.** Tirzepatide significantly reduces white adipose tissue depots in females (unpaired t-tests). **L.** Tirzepatide significantly reduces hepatic triglyceride content in treated females (unpaired t-test). **M.** Treatment reduces IL-6 and TNFα serum levels in females, while levels of IL-1β, IL-10 and MCP-1 remain unaffected (unpaired t-test). Data show mean ± SEM with individual data points. ∗P < 0.05, ∗∗P < 0.01, ∗∗∗P < 0.001.

Male rats receiving repeated tirzepatide showed sustained reductions in alcohol consumption (treatment F_1,18_ = 20.66, P < 0.001, interaction F_5,90_ = 1.39, P = 0.234; Fig. 4A), with consistent decreases compared to vehicle throughout the study period. Alcohol preference was also attenuated (Supplementary Figure S9A). Water intake remained unaffected by treatment (Supplementary Figure S9B), while tirzepatide significantly reduced total fluid intake, food consumption and body weight (Supplementary Figure S9C–E) across the experimental timeline.

Female rats demonstrated similar sustained responses during repeated treatment. Tirzepatide produced comparable reductions in alcohol intake (treatment F_1,18_ = 84.59, P < 0.001, interaction F_5,90_ = 1.24, P = 0.296; Fig. 4B) that persisted throughout the two-week period. As observed in males, alcohol preference was decreased (Supplementary Figure S9F). Water consumption remained stable (Supplementary Figure S9G), while total fluid intake, food intake and body weight decreased significantly under tirzepatide administration (Supplementary Figure S9H–J).

Direct comparison revealed no significant sex differences in treatment response (sex F_1,18_ = 1.60, P = 0.222, interaction F_5,90_ = 0.63, P = 0.679), with females showing 63.4 ± 4.1% reductions compared to 54.3 ± 7.1% in males (Fig. 4C).

### Tirzepatide improves metabolic and inflammatory markers in alcohol-consuming rats across both sexes

Long-term alcohol use can lead to fatty liver disease, metabolic syndrome, and systemic inflammation, conditions that complicate treatment.^37^, ^38^, ^39^ GLP-1R agonists and tirzepatide have shown effects on metabolic liver disease and inflammatory processes.^58^, ^59^, ^60^, ^61^ Given the notable reduction in body weight we observed during the repeated treatment (Exp 11), we additionally explored whether tirzepatide might also affect metabolic and inflammatory parameters in alcohol-consuming animals. Preliminary insights on these effects might inform whether tirzepatide could serve dual therapeutic roles, treating both alcohol use behaviours and the complications that often accompany AUD.

In male rats, body weight changes diverged significantly between treatment groups (treatment F_1,18_ = 6.39, P = 0.021, interaction F_11,198_ = 95.70, P < 0.001; Fig. 4D), with vehicle-treated rats gaining weight while tirzepatide-treated rats lost weight. Percentage body weight analysis showed more pronounced between-group differences (F_1,18_ = 181.80, P < 0.001, interaction F_11,198_ = 97.35, P < 0.001; Fig. 4E), with the tirzepatide group showing an average decrease of 8.5 ± 0.6% and vehicle group an average increase of 3.0 ± 0.2%. Tissue-specific analysis revealed selective effects of this weight reduction. Tirzepatide treatment did not significantly alter gastrocnemius muscle (t_18_ = 1.32, P = 0.204; Fig. 4F) or iBAT mass (t_18_ = 1.22, P = 0.238) compared to vehicle. However, tirzepatide significantly reduced white adipose tissue (WAT) deposits across multiple locations: sWAT (t_18_ = 3.10, P = 0.006), gWAT (t_18_ = 2.12, P = 0.048), and rpWAT (t_18_ = 3.19, P = 0.005). Tirzepatide also decreased liver weight in treated males (t_18_ = 2.15, P = 0.045; Fig. 4F), with accompanying reductions in hepatic triglyceride content (t_18_ = 3.70, P = 0.002; Fig. 4G). Tirzepatide significantly reduced serum concentrations of pro-inflammatory cytokines IL-6 (t_18_ = 2.49, P = 0.023) and TNFα (t_18_ = 2.17, P = 0.043; Fig. 4H) in male rats, while levels of IL-1β, IL-10, and MCP-1 remained unaffected.

Female rats exhibited comparable metabolic and inflammatory responses to tirzepatide treatment. Body weight changes showed similar divergence between treatment groups (F_1,18_ = 12.93, P = 0.002, interaction F_11,198_ = 39.48, P < 0.001; Fig. 4I), with tirzepatide inducing weight reduction while vehicle-treated females gained weight. Percentage body weight changes revealed an 8.8 ± 0.6% decrease in tirzepatide-treated females compared to a 2.3 ± 0.5% increase in vehicle-treated counterparts (F_1,18_ = 136.70, P < 0.001, interaction F_11,198_ = 40.17, P < 0.001; Fig. 4J). As observed in males, tirzepatide's effects on body composition in females were tissue-specific. Tirzepatide preserved gastrocnemius muscle (t_18_ = 2.02, P = 0.058; Fig. 4K) and iBAT mass (t_18_ = 0.59, P = 0.564), while significantly reducing WAT deposits across all measured depots: sWAT (t_18_ = 4.49, P < 0.001), gWAT (t_18_ = 6.38, P < 0.001), and rpWAT (t_18_ = 3.84, P = 0.001). Tirzepatide significantly reduced liver weight in treated females (t_18_ = 2.58, P = 0.019; Fig. 4K), with concomitant decreases in hepatic triglyceride content (t_18_ = 2.99, P = 0.008; Fig. 4L). The inflammatory profile mirrored that observed in males, with significant reductions in serum IL-6 (t_18_ = 2.62, P = 0.017) and TNFα (t_18_ = 2.16, P = 0.044; Fig. 4M) levels following tirzepatide treatment.

### Ex vivo electrophysiological recordings in alcohol-naïve mice identified lateral septum as a possible target for tirzepatide's neural effects

Having preserved brain tissue from the repeated tirzepatide and alcohol drinking experiment (Exp 11), we required a systematic rationale for selecting which region to subject to proteomic analysis. An ex vivo electrophysiological screening paradigm (Exp 12) was therefore implemented in which alcohol-naïve mice received acute tirzepatide administration (30 nmol/kg, SC), with evoked synaptic responses assessed 24 h post-treatment across multiple reward-related^9^^,^^40^ circuits: NAc core/shell, mPFC, DLS, DMS, and LS. This screening approach enabled identification of the brain region exhibiting the most pronounced neuroplastic alterations, thereby informing the subsequent proteomic investigation of corresponding tissue from Exp 11.

**Fig. 5:**
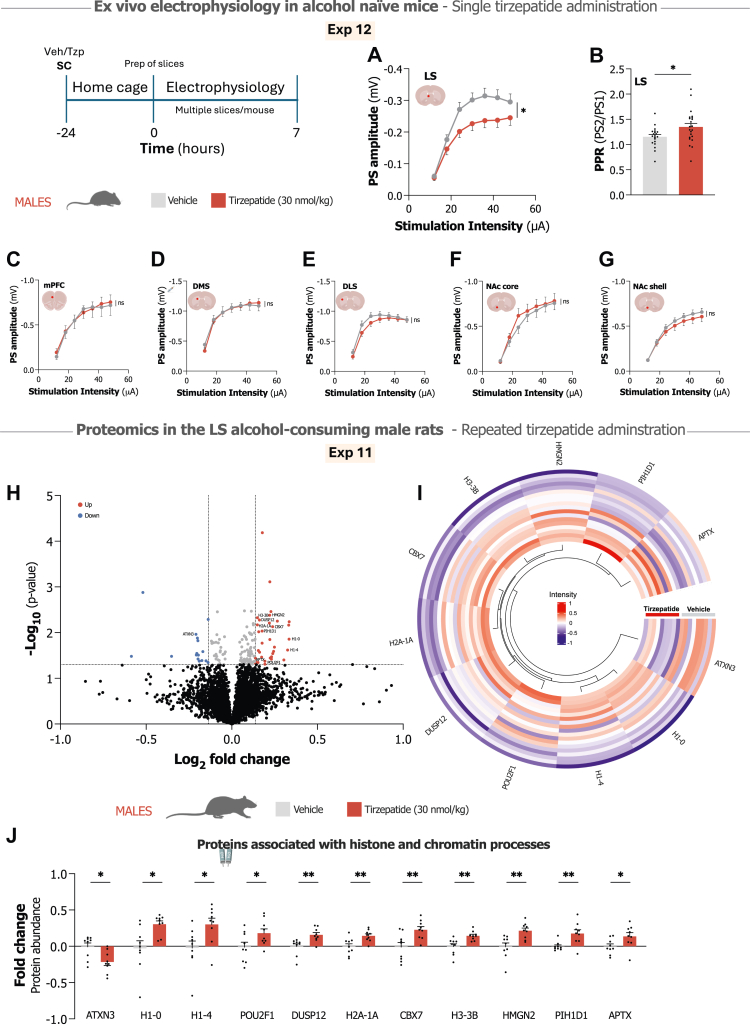
**Electrophysiological effects of tirzepatide in alcohol-naïve male mice and proteomic alterations following repeated tirzepatide administration in alcohol-consuming male rats. A.** Input-output curves derived from ex vivo brain slice field recordings from male mice demonstrate tirzepatide-induced reduction in population spike (PS) amplitude within the lateral septum (LS) (n = 28–30/group, repeated measures two-way ANOVA). **B.** Paired-pulse ratio (PPR, PS2/PS1) measurements in the LS reveal significant effects in tirzepatide-treated mice compared to the vehicle group (n = 20–22/group, unpaired t-test). **C–G.** Input-output curves in the medial prefrontal cortex (mPFC), dorsomedial striatum (DMS), dorsolateral striatum (DLS), nucleus accumbens (NAc) core, and NAc shell demonstrate region-specific electrophysiological responses, with no statistically significant differences observed in these regions following tirzepatide treatment (n = 9–30/group, repeated measures two-way ANOVA). **H.** Volcano plot visualises the proteomics data in the LS of alcohol-consuming male rats following repeated tirzepatide (30 nmol/kg) administration, showing the differential protein expression profile. **I.** Circular heatmap visualises differentially expressed proteins associated with histone and chromatin processes following repeated tirzepatide treatment in alcohol-drinking male rats. **J.** Quantification shows fold changes in specific proteins associated with histone and chromatin processes comparing tirzepatide treatment against vehicle, with significant differences observed in multiple proteins (n = 9/group, Welch's t-test, ∗p < 0.05, ∗∗p < 0.01).

Analysis of the LS recordings revealed that tirzepatide exposure 24 h earlier produced a sustained suppression of evoked field potentials (F_1,56_ = 4.74, P = 0.034; Fig. 5A), which was concomitant with a significant increase in paired-pulse ratio (t_40_ = 2.32, P = 0.026; Fig. 5B), indicative of a decreased probability of neurotransmitter release. This suggests tirzepatide induced lasting presynaptic modifications within the LS region. In contrast, we observed no sustained differences when comparing tirzepatide-exposed mice with vehicle-treated mice in other reward-related circuits, including the mPFC (F_1,19_ = 0.01, P = 0.924), DMS (F_1,34_ = 0.01, P = 0.907), DLS (F_1,58_ = 1.06, P = 0.308), NAc core (F_1,19_ = 0.26, P = 0.617), or NAc shell (F_1,58_ = 0.48, P = 0.490; Fig. 5C–G).

### Proteomic analysis of the LS using brain samples from alcohol-consuming male rats receiving repeated tirzepatide reveals chromatin regulatory proteins as potential targets of tirzepatide's effects

Based on electrophysiology results identifying the LS as a region showing sustained responses to tirzepatide, we conducted a global quantitative proteomic analysis of the LS using tissue from male alcohol-drinking rats that received repeated tirzepatide treatment (Exp 11). We identified 4359 distinct proteins using TMT mass spectrometry, with statistical analysis showing 51 proteins differentially expressed between tirzepatide and vehicle groups (Fig. 5H, Supplementary Table S1). Tirzepatide upregulated 35 proteins and downregulated 16 proteins compared to vehicle.

Gene Ontology analysis identified several functional categories among the differentially expressed proteins (DEPs). We found proteins previously linked to alcohol consumption, including peroxisomal trans-2-enoyl-CoA reductase (PECR), midkine (MDK), acetyl-CoA acyltransferase 2 (ACAA2), ATP-binding cassette subfamily G member 2 (ABCG2), and reticulon 1 (RTN1). Tirzepatide also affected proteins involved in neurotransmission, such as solute carrier family 6 member 6 (SLC6A6), reticulon 3 (RTN3), proline-rich transmembrane protein 2 (PRRT2), alpha-aminoadipic semialdehyde synthase (AASS), and microtubule-associated protein 1A (MAP1A). We also detected changes in neuroinflammation-related proteins, including PECR, signal regulatory protein alpha (SIRPA), and leucine-rich repeat containing 14 (LRRC14).

The DEPs also included several histone proteins: H1-0, H1-4, H2A-1A, and H3-3B. Given that histone modifications and chromatin remodelling represent important epigenetic mechanisms involved in addiction pathophysiology,^10^^,^^11^ we focused detailed analysis on GO terms related to histone and chromatin processes. Cross-referencing with the UniProtKB database^53^ and existing literature revealed a total of 11 proteins associated with histone or chromatin regulatory functions, visualised as a heat map^62^ in Fig. 5I (with references listed in Supplementary Table S1). Statistical analysis showed significant differences between tirzepatide and vehicle groups in these 11 proteins: ataxin-3 (ATXN3; t_15.9_ = 2.88, P = 0.011), histone H1-0 (t_10.7_ = 2.93, P = 0.014), histone H1-4 (t_15.7_ = 2.50, P = 0.024), POU domain class 2 transcription factor 1 (POU2F1; t_15.7_ = 2.14, P = 0.049), dual specificity protein phosphatase 12 (DUSP12; t_15.5_ = 3.24, P = 0.005), histone H2A-1A (t_14.2_ = 3.17, P = 0.007), chromobox protein homologue 7 (CBX7; t_14.2_ = 3.12, P = 0.007), histone H3-3B (t_12.5_ = 3.43, P = 0.005), high mobility group nucleosome-binding domain-containing protein 2 (HMGN2; t_14.2_ = 3.41, P = 0.004), PIH1 domain-containing protein 1 (PIH1D1; t_10.5_ = 3.19, P = 0.009), and aprataxin (APTX; t_14.0_ = 2.22, P = 0.043) (Fig. 5J).

## Discussion

Here we demonstrate that tirzepatide, a dual GLP-1R/GIPR agonist, affects alcohol intake and alcohol-related responses across both sexes in rodents. Our findings reveal that tirzepatide attenuates alcohol-induced dopamine signalling, reduces alcohol consumption, and suppresses relapse-like behaviours. We also observed changes in metabolic and inflammatory parameters linked to alcohol use. Through electrophysiological and proteomic approaches, we identified the LS as what may be an important neuroanatomical substrate, with proteomic data suggesting potential epigenetic involvement in tirzepatide's effects. These findings indicate that tirzepatide may represent a promising therapeutic candidate for AUD, potentially addressing both drinking behaviour and the physiological consequences of alcohol intake that likely contribute to poor treatment outcomes and relapse vulnerability.

Our initial investigation examined tirzepatide's effects on reward-related responses and revealed significant suppression of alcohol-induced dopamine processing (Exp 1–6). Tirzepatide consistently reduced alcohol-induced locomotor stimulation (Exp 1), place preference (Exp 3), and accumbal dopamine release (Exp 5–6) in male mice across these paradigms. This modulation is noteworthy given that alcohol-induced dopamine release in the NAc appears to contribute to consummatory behaviours and likely represents a risk factor for AUD development.^5^, ^6^, ^7^, ^8^ Perhaps most compelling was our observation that tirzepatide suppressed alcohol-induced dopamine release regardless of whether alcohol was administered systemically (Exp 5) or perfused locally within the NAc itself (Exp 6). This suggests tirzepatide may directly influence the reward circuitry, though the precise mechanisms warrant further investigation. These findings align with previous work showing that GLP-1R agonists affect dopaminergic reward pathways across different substances of abuse.^23^, ^24^, ^25^^,^^27^^,^^63^, ^64^, ^65^, ^66^, ^67^, ^68^, ^69^

The observed effects on reward processing appeared to translate into substantial reductions in alcohol-drinking behaviour. Tirzepatide consistently decreased voluntary alcohol intake and alcohol preference across both sexes in multiple experimental paradigms (Exp 7–11). Single administration produced dose-dependent reductions in alcohol consumption in rats (Exp 7–8) while also suppressing binge-like drinking in mice (Exp 9), suggesting cross-species effectiveness. These observations extend what we and others have seen with GLP-1R agonists, where similar alcohol intake-suppressing effects have emerged across various preclinical models.^21^^,^^22^^,^^25^^,^^26^^,^^28^^,^^70^, ^71^, ^72^, ^73^ Another interesting observation was that the repeated tirzepatide administration (Exp 11) appeared to maintain its suppressive effects more consistently than what we saw in our previous work with semaglutide,^25^ though we acknowledge this comparison spans different studies with inherent limitations. The clinical landscape looks increasingly promising. Early studies show that GLP-1R agonists reduce alcohol consumption in humans,^29^, ^30^, ^31^, ^32^ suggesting our preclinical models might capture clinically relevant mechanisms. Furthermore, a recent case–control study found reduced alcohol consumption in patients with obesity receiving tirzepatide,^74^ providing additional support for potential therapeutic applications. While these observations emerge from metabolic treatment contexts rather than addiction-focused studies, they provide what appears to be real-world validation of our experimental findings. Since tirzepatide and other GLP-1R agonists already have clinical approval for type 2 diabetes and obesity, a solid foundation exists for further AUD investigation. This practical advantage appears to have facilitated current clinical initiatives, with multiple studies now exploring incretin agonists potential as addiction treatment.

Our findings on relapse-like behaviours (Exp 4 and 10) add further relevance to these clinical applications. Relapse remains a clinical challenge in AUD and effective treatment options remain scarce.^9^^,^^55^^,^^56^ We found that tirzepatide blocks ADE (Exp 10), a model of relapse-like drinking,^47^ which aligns with our earlier work with liraglutide and semaglutide.^25^^,^^70^ Tirzepatide also attenuated cue-induced place preference following a period of no exposure to alcohol or alcohol-paired context and cues (Exp 4), suggesting possible effects on drug memory and cue reactivity processes.^55^^,^^56^ Existing literature indicates that GLP-1R agonists also suppress reinstatement for cocaine,^67^ nicotine,^68^ and opioids,^69^ further supporting a potential effect on relapse-like behaviours. Furthermore, our findings seem translationally promising, given recent reports that semaglutide reduces alcohol cravings in humans^29^ and that exenatide diminishes cue reactivity in patients with AUD.^30^ Both anecdotal and research-based observations also support this interpretation, with reports of reduced cravings for food and substances during GLP-1R agonist treatment.^74^, ^75^, ^76^ As both cravings and environmental cues often precipitate relapse,^55^^,^^56^ our findings further support the emerging evidence that incretin agonists could help mitigate relapse risk.

The repeated administration (Exp 11) study also revealed effects on metabolic and inflammatory parameters that warrant further investigation. Given planned clinical trials exploring tirzepatide and semaglutide for alcohol liver disease and patients with AUD and metabolic comorbidities (ClinicalTrials.gov identifiers: NCT06546384, NCT06409130, and NCT07046819), these findings could take on added significance. While incretin agonists demonstrate established benefits in metabolic and inflammatory conditions,^58^, ^59^, ^60^, ^61^ their effects in long-term alcohol-consuming populations remain less well characterised. The question is whether they maintain these effects when alcohol consumption is involved. Our findings suggest they might. Repeated tirzepatide treatment reduced body weight, adipose tissue mass, liver weight, and hepatic triglyceride content, and also decreased pro-inflammatory cytokines (IL-6, TNFα) in both male and female alcohol-consuming rats. These observations suggest tirzepatide may offer therapeutic potential for alcohol-related complications such as fatty liver disease, a finding with clear implications for patients managing concurrent AUD and metabolic disorders. Early clinical evidence aligns with this. Both exenatide and semaglutide reduce alcohol consumption in individuals with obesity,^29^^,^^30^ suggesting the dual effects on metabolism and alcohol intake we observe preclinically may hold therapeutic value in relevant patient populations. That said, the concomitant reductions in food intake and body weight observed with tirzepatide warrant consideration, particularly for non-obese individuals seeking addiction treatment. However, the high prevalence of metabolic dysfunction in AUD populations suggests this dual effect may prove therapeutically relevant rather than simply problematic, though careful dose optimisation would be necessary. Clinical studies specifically in alcohol-using populations will be necessary to validate these applications. Our alcohol consumption studies (Exp 7–11) demonstrated generally consistent effects across sexes at the 30 nmol/kg dose used in most paradigms. In the acute IA2BC studies (Exp 7 and 8) where we tested both 10 and 30 nmol/kg, males responded at both doses while females showed effects only at the higher dose. Supportingly, sex-divergent effects of GLP-1R agonists on alcohol intake have been observed previously.^24^^,^^25^^,^^77^^,^^78^ Several factors might contribute to this observation. Gonadal hormones, particularly oestrogen, influence GLP-1R function^79^, ^80^, ^81^, ^82^ and alcohol-related behaviours,^78^^,^^83^^,^^84^ and we have previously found that long-term alcohol consumption produces sex-dependent changes in GLP-1R expression, with elevated receptor levels in the NAc and LS in high-consuming males that were not observed in females.^23^^,^^24^ Whether these tentative differences interact with tirzepatide's dual receptor mechanism, or whether GIPR regulation shows similar sex-dependent patterns, remains an open question that future systematic dose–response and mechanistic studies would need to address, preferably in both rats and mice.

Given the overall consistent effects across sexes with the 30 nmol/kg dose, subsequent mechanistic experiments focused on male rodents to identify potential brain regions mediating tirzepatide's effects on alcohol-related responses. We started with electrophysiological recordings across several reward-related circuits (Exp 12). To examine tirzepatide's long-lasting effects, we assessed putative neuroplasticity 24 h after a single injection. Neurotransmission was similar independent of treatment across most brain regions, but the LS showed sustained synaptic depression associated with decreased probability of presynaptic neurotransmitter release. This finding is notable given the region's established role in reward processing and drug-seeking behaviours.^40^^,^^85^^,^^86^ Moreover, the LS is positioned strategically near the subfornical organ, a circumventricular organ that might serve as an access point for peripherally administered incretin agonists,^87^ and maintains direct connections to the NAc and ventral tegmental area,^40^^,^^85^^,^^86^ allowing it to potentially influence mesolimbic dopamine signalling.^88^ Both GLP-1R^89^^,^^90^ and GIPR^91^^,^^92^ are expressed in the LS, and peripherally administered GLP-1R agonists can indeed reach this region.^21^^,^^93^^,^^94^ Previous preclinical work has also demonstrated LS involvement in GLP-1R-mediated reward processing for both alcohol^21^^,^^24^ and cocaine-related behaviours,^63^^,^^95^ as well as regulation of alcohol-induced dopamine release in the NAc.^24^ Of note, a clinical study with GLP-1R agonist exenatide demonstrated reduced septal cue reactivity to alcohol in patients with AUD.^30^ This convergent evidence led us to hypothesise that the LS might serve as a neuroanatomical substrate mediating tirzepatide's effects on mesolimbic dopamine signalling and alcohol-related responses.

To gain some insights into potential LS-related molecular mechanisms, we conducted a proteomic analysis of LS tissue from alcohol-consuming male rats from the repeated tirzepatide treatment experiment (Exp 11). This revealed differential expression of several histone and chromatin regulatory proteins, including specific histone proteins (H1-0, H1-4, H2A-1A, H3-3B). These findings seemed noteworthy because histones regulate gene expression and serve as essential components of chromatin architecture.^10^^,^^13^ Given that alcohol exposure can cause histone modifications, such as acetylations and methylations, which may contribute to addiction pathophysiology,^10^^,^^11^^,^^96^^,^^97^ our results suggest tirzepatide might influence epigenetic mechanisms involved in alcohol drinking behaviours. Recent clinical research with semaglutide in obesity has identified similar proteomic changes in blood samples, including proteins linked to substance use disorders, such as histones.^98^ These findings hint that GLP-1R-based therapeutics may engage epigenetic mechanisms, tentatively explaining their broad efficacy across multiple disorders. However, future studies will need to investigate these potential mechanisms more thoroughly.

It should, however, be noted that our study has several limitations that should be acknowledged. First, we exclusively used male mice in our behavioural, microdialysis and electrophysiological experiments, and male rats for proteomic analysis. While this approach allowed us to compare our results with established literature and avoided the resources needed to establish and validate models across sexes, it limits the generalisability of our findings. Although acute alcohol stimulation and GLP-1/GIP receptor agonist effects are expected to be similar between sexes, sex differences in addiction-related behaviours are well-documented.^83^^,^^84^ Future studies should explicitly include female subjects to ensure these promising effects of tirzepatide translate across sexes, particularly given that AUD affects both men and women.^99^^,^^100^ While our experimental design excluded potential confounding factors such as sedation, anhedonia, and malaise (as tirzepatide did not affect baseline locomotor activity (Exp 1 and the locomotion dose–response experiment), dopamine levels per se (Exp 5), or kaolin consumption (intake dose–response experiment)), we did not specifically assess anxiety-like behaviours which could influence alcohol-related responses. The absence of alcohol-exposed mice in the electrophysiological study is a potential confounding factor that future studies should assess, as differences in respective brain regions between groups may differ when alcohol is involved. However, it was recently shown that LS GLP-1R also modulates neural activity in long-term alcohol consuming animals,^24^ so some similarities would be expected. Furthermore, our proteomic analysis was limited to a single brain region and could not distinguish whether tirzepatide directly induced proteomic alterations or if these changes emerged as a consequence of decreased alcohol intake. Future studies should address this through targeted mechanistic investigations to characterise the molecular pathways linking receptor activation to downstream neuroadaptations in the LS. Exploring LS connectivity to clarify the relative contributions of specific neural pathways to tirzepatide's effects on reward processing and alcohol consumption is also warranted. A more comprehensive direct comparison of tirzepatide versus semaglutide within the same study would furthermore elucidate their differential efficacy and neurobiological mechanisms in modulating reward circuitry. Despite these limitations, our findings suggest that tirzepatide influences the reward circuitry and may serve as a therapeutic candidate for AUD and related alcohol-related conditions.

In summary, our findings indicate that tirzepatide influences alcohol-related responses in ways that appear to have clinical potential. Tirzepatide consistently reduced alcohol intake across different drinking paradigms and both sexes without signs of tolerance development. Perhaps more significantly, tirzepatide's effects on relapse behaviours suggest it might help decrease relapse vulnerability, a finding that could prove important for therapeutic applications. The mesolimbic reward effects offer insights into tirzepatide's possible mode of action. Our data suggest tirzepatide influences dopaminergic reward processes to suppress alcohol-drinking behaviours. The LS findings add another piece to this puzzle, offering some initial clues about a neural substrate where dual incretin receptor signalling might exert these effects. These results, combined with tirzepatide's existing clinical approval, position this dual incretin agonist as a promising therapeutic candidate for AUD and alcohol-related diseases, that warrants clinical investigation.

## Contributors

Conceptualisation: CEE, LA, MFL, HCB & EJ. Methodology: CEE, LA, AB, IWA, HCB & EJ. Investigation: CEE, LA, SG, SA, TAE, YG, AT, MV & CA. Formal analysis: CEE, LA, SG, SA, TAE, YG, AT, MV, MFL, HCB & EJ. Data curation: CEE, LA, SG, SA, TAE, YG, AT, MV & EJ. Writing—original draft: CEE. Writing—review & editing: CEE, LA, SG, SA, TAE, YG, AT, MV, CA, AB, IWA, MFL, HCB & EJ. Visualisation: CEE. Project administration: CEE & EJ. Resources: LA, AB, IWA, MFL, HCB & EJ. Funding acquisition: CEE, LA, MV, IWA, HCB & EJ. Validation: CEE, LA, MFL, HCB & EJ. Data verification: CEE, LA, MFL, HCB & EJ. All authors have read and approved the final version of the manuscript.

## Data sharing statement

Data collected for the study will be made available and shared with others though contact at the following address: elisabet.jerlhag@pharm.gu.se. The data will be shared with researchers who want to do additional analysis of the data and therefore the data will be shared after approval of a proposal, and with a signed data access agreement. The MS proteomics data have been deposited to the ProteomeXchange Consortium (http://proteomecentral.proteomexchange.org) via the PRIDE partner repository with the data set identifier PXD063324.

## Declaration of interests

Authors declare that they have no competing interests.
